# Selective Surface Electrostimulation of the Denervated Zygomaticus Muscle

**DOI:** 10.3390/diagnostics11020188

**Published:** 2021-01-28

**Authors:** Dirk Arnold, Jovanna Thielker, Carsten M. Klingner, Wiebke Caren Puls, Wengelawit Misikire, Orlando Guntinas-Lichius, Gerd Fabian Volk

**Affiliations:** 1 Facial-Nerve-Center Jena, Jena University Hospital, 07740 Jena, Germany; d.arnold@uni-jena.de (D.A.); jovanna.thielker@med.uni-jena.de (J.T.); CARSTEN.KLINGNER@med.uni-jena.de (C.M.K.); wiebke.pittack-puls@t-online.de (W.C.P.); Wengelawit@gmx.net (W.M.); orlando.guntinas@med.uni-jena.de (O.G.-L.); 2 Institute of Zoology and Evolutionary Research, Friedrich-Schiller-University Jena, 07743 Jena, Germany; 3 Department of Otorhinolaryngology, Jena University Hospital, 07740 Jena, Germany; 4 Department of Neurology, Jena University Hospital, 07740 Jena, Germany

**Keywords:** electrostimulation, surface electrodes, facial paralysis, facial palsy, zygomaticus muscle, muscle atrophy, reinnervation

## Abstract

This article describes a first attempt to generate a standardized and safe selective surface electrostimulation (SES) protocol, including detailed instructions on electrode placement and stimulation parameter choice to obtain a selective stimulation of the denervated zygomaticus muscle (ZYG), without unwanted simultaneous activation of other ipsilateral or contralateral facial muscles. Methods: Single pulse stimulation with biphasic triangular and rectangular waveforms and pulse widths (PW) of 1000, 500, 250, 100, 50, 25, 15, 10, 5, 2, 1 ms, at increasing amplitudes between 0.1 and 20 mA was performed. Stimulations delivered in trains were assessed at a PW of 50 ms only. The stimulation was considered successful exclusively if it drew the ipsilateral corner of the mouth upwards and outwards, without the simultaneous activation of other ipsilateral or contralateral facial muscles. I/t curves, accommodation quotient, rheobase, and chronaxie were regularly assessed over 1-year follow-up. Results: 5 facial paralysis patients were assessed. Selective ZYG response in absence of discomfort and unselective contraction of other facial muscle was reproducibly obtained for all the assessed patients. The most effective results with single pulses were observed with PW ≥ 50 ms. The required amplitude was remarkably lower (≤5 mA vs. up to 15 mA) in freshly diagnosed (≤3 months) than in long-term facial paralysis patients (>5 years). Triangular was more effective than rectangular waveform, mostly because of the lower discomfort threshold of the latter. Delivery of trains of stimulation showed similar results to the single pulse setting, though lower amplitudes were necessary to achieve the selective ZYG response. Initial reinnervation signs could be detected effectively by needle-electromyography (n-EMG). Conclusion: It is possible to define stimulation parameters able to elicit an effective selective stimulation of a specific facial muscle, in our case, of the ZYG, without causing discomfort to the patient and without causing unwanted unspecific reactions of other ipsilateral and/or contralateral facial muscles. We observed that the SES success is strongly conditioned by the correct electrode placement, which ideally should exclusively interest the area of the target muscles and its immediate proximity.

## 1. Introduction

Facial paralysis is a neurological condition in which the facial nerve (cranial nerve VII) functionality is completely lost, i.e., the innervated facial muscles are completely denervated, in contrast to facial paresis, a condition in which some motor activity can still be detected. Facial palsy is a definition that does not describe the severity of the nerve damage, thus can refer to either facial paralysis or paresis. Facial palsy can have an idiopathic origin or be caused by traumas, infections, tumors, surgical interventions, or congenital syndromes [1,2,3,4]. Central lesions like intracranial tumors or strokes result in central facial palsy, which only affect the second motor neuron indirectly. This article focuses exclusively on the peripheral facial palsy, a form affecting the ipsilateral mimic muscles, and, in particular, the eyelids, the forehead, and the mouth. It may associate with sensory and autonomic disorders, depending on the exact location of the lesion. Since facial palsy causes a noticeable deformity and severely impairs non-verbal communication, it has a relevant emotional impact on the patients, and may lead to self-isolation and reduced self-esteem [5,6]. In 2013 Hohman et al. [7] reported that in the 1810 patients they assessed and who developed facial palsy between 2002 and 2012, 5.7% were diagnosed with a iatrogenic origin. In their work, they confirmed that idiopathic facial palsy, better known as Bell’s palsy, with an annual prevalence of about 25 cases per 100,000 [1], was the most common cause (60%) of peripheral facial palsy [4]. While, in the majority of the cases, idiopathic facial palsy tends to show a complete recovery within 3–9 weeks, this feature is not shared by iatrogenic or traumatic forms. If facial palsy persists for longer than three months, it is known as chronic facial palsy and often requires surgical treatment for effective symptom mitigation, such as surgical reinnervation [8,9,10,11,12,13,14], even if surgical approaches are almost never able to ensure a complete recovery [15]. For instance, surgical reinnervation effectiveness is often reduced by the occurrence of misdirected axon regeneration causing synkinesis and the consequent so called post-paralytic facial syndrome [16], or delayed by the low rate of nerve grow (1 mm/d) in case of extended injuries [17,18].

Electrostimulation is a potential new therapeutic approach that has been increasingly investigated in the last decades, with clinical implications for the ENT field in general, and for the facial paralysis treatment in particular. For instance, some recent studies have proposed to use facial electromyography (EMG) to detect the physiological activation of the contralateral healthy facial muscles and use it to trigger functional electrical stimulation on the ailing side with the goal to regain symmetry at rest and during facial movements [19]. Because of the obvious difficulties in designing an implantable system for the treatment of facial palsy, and its potential drawbacks, such as invasiveness, the majority of the studies is currently confined to animal models. On the contrary, non-invasive delivery of selective surface electrostimulation (SES) may become a safer and more easily implementable solution for the near future [15,20]. In the majority of incomplete facial palsy cases, irrespective of their etiology, the affected facial muscles remain functional, in spite of atrophy. On the contrary, complete facial paralysis is expected to severely worsen the atrophy process in a very short time after onset, and thus to reduce or even abolish the functionality of the affected muscles. Previous studies on the therapeutic use of electrostimulation [21,22] have provided initial evidence that SES can effectively counteract atrophy, suggesting that it has the potentiality to slow down if not even to prevent the reduction of muscle volume and functionality in patients suffering from facial paralysis [21,22,23,24,25,26]. A major issue against a widespread use of SES in the treatment of facial paralysis is the difficulty to determine a combination of parameters capable to generate a selective stimulation delivered by surface electrodes. The possibility that electrostimulation of denervated muscles could prevent or at least slow down reinnervation has been a matter of dispute in the last years. Another issue preventing its use in standard therapy has been the potential risk of inducing synkinetic rather than normal reinnervation in the targeted muscles, although recently, evidence has been published against both the aforementioned concerns [20].

This article describes our first attempt to assess the most effective stimulation parameters for a safe SES that can be implemented as daily home training by the patients suffering from facial paralysis. We chose the ZYG as target for our study because of its size, position, and clearly discernable response to the stimulation. In addition, the electrode placement could be easily corrected whenever an unspecific response of other facial muscles is observed.

## 2. Methods

### 2.1. Study Characteristics

The data presented in this article were generated between June 2018 and July 2019 in the Facial Nerve Center of the ENT Department of the Jena University Hospital within a longitudinal, open-label, prospective, monocentric, case-series-based, proof of principle clinical investigation, approved by the ethics committee in 2018 (application number 5505-03/18). Informed consent was obtained from all participants, in compliance with the Declaration of Helsinki as amended in Fortaleza (2013). The study was registered in the German Clinical Trials Register (Deutsches Register Klinischer Studien; DRKS).

### 2.2. Study Timeline

The study foresaw 12 visits, three of which (the 2nd, the 3rd and the 5th visit) were conducted over the phone. 4/5 (80%) patients completed all of them (every 4 weeks). Patient DFP0101001 left the study after the completion of the 8th follow-up because of clinically relevant reinnervation.

### 2.3. Study Aims

The study had two separate aims: (a) to generate a standardized SES protocol, including detailed instructions on the most effective electrode placement and stimulation parameter choice in order to obtain a selective stimulation of the denervated ZYG, without the simultaneous activation of other ipsi- and/or contralateral facial muscles, and not causing discomfort to the patient; (b) to assess the effects of daily home SES using the protocol defined as depicted above. This article exclusively focuses on the first aim. A future publication is planned to depict the results concerning the second goal.

### 2.4. Population

Five adults (four females) were recruited, who were diagnosed with unilateral facial paralysis confirmed by needle-EMG (n-EMG). Their average age was 43.0 ± 12.5 [range: 29–59 years]. Table 1 displays a detailed demographic and facial paralysis history analysis of the recruited patients. While in 4/5 (80%) of the cases the denervation had an iatrogenic origin and was characterized by a sudden onset, in 1/5 cases (20%), it was of tumorigenic origin and progressive (patient DFP0101004). This latter patient was the only one having performed 1-year electrostimulation at home before being enrolled in our study. In his case, pre-study home-training included 30–50 min sessions twice per day with an amplitude up to 22 mA, delivered with a PW of 150 ms and biphasic triangular impulses by means of the stimulation device Paresestim (Krauth + Timmermann, Hamburg, Germany). Patient DFP0101001 was withdrawn from the study at the 8th follow-up visit, following clinically relevant ZYG reinnervation upon facial nerve reinnervation surgery. Patients DFP0101002 and −005 showed clinically relevant ZYG reinnervation with macroscopic voluntarily contractions at the last follow-up visit, while no reinnervation could be detected for the entire duration of the follow-up period in patient DFP0101003, suffering from chronic facial paralysis for about 16 years. Single fiber activity, compatible with non-clinically relevant reinnervation could be detected in patient DFP0101004, suffering from facial paralysis for about 6 years after 8 m in the study.

### 2.5. Electrode Placement

**Figure 1:**
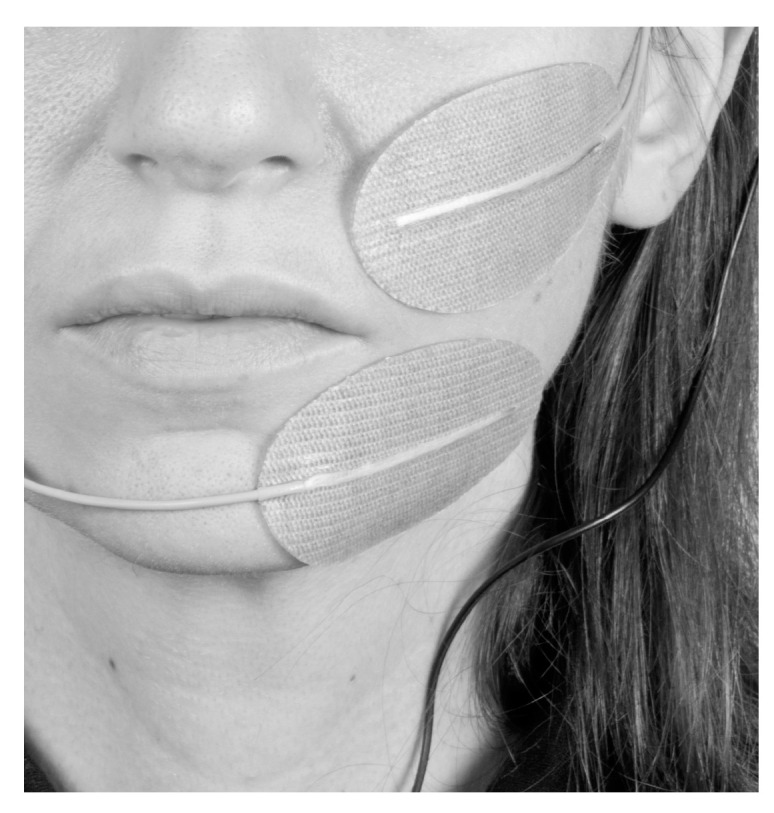
Example of how the electrode placement was conducted during the study (from the patient’s diary specifically generated for the study).

With the patient in upright sitting position, two 60 × 40 mm oval surface electrodes (Krauth+Timmermann, Hamburg, Germany) were placed in correspondence with the paralyzed ZYG as close as possible to the mouth corner in order to prevent unspecific stimulation of other facial muscles (Figure 1). STMIsola (BIOPAC Systems, Inc., Essen, Germany) connected to the PowerLab (ADInstruments, Sydney, Australia) system was used to deliver the stimulation. The upper electrode was always the cathode and the lower the anode. The muscle was stimulated bipolarly, without the use of a reference electrode to selectively limit the electrical field to the area of the ZYG.

### 2.6. Stimulation Parameters

**Figure 2:**
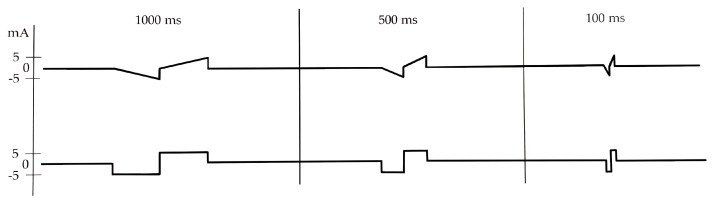
Biphasic single pulse stimulation pattern with a PW of 1000, 500 and 100 ms, delivered with triangular (above) or rectangular waveform (below). ms = milliseconds; mA = milliampere.

At each session at the hospital, the patient underwent single pulse stimulation to determine the “stimulation threshold”. Biphasic triangular and rectangular waveforms were tested, with a PW of 1000, 500, 250, 100, 50, 25, 15, 10, 5, 2, 1 ms, at increasing amplitudes between 0.1 and 20 mA (Figure 2). The amplitude was increased stepwise by 0.5 mA until a visually detectable movement of the mouth corner was observed. The “stimulation threshold” was recorded based on the independent observation of the ZYG response to the stimulation by two investigators. In addition to single pulse stimulation, biphasic triangular waveform trains of 20 pulses each, with a PW of 50 ms, a frequency of 7 Hz, and a pause of 50 ms, and biphasic rectangular waveform trains with a PW of 50 ms and a frequency of 7 Hz were delivered. The average duration of each session was 30 min.

### 2.7. Outcome Measures

ZYG SES was considered successful only if it drew the ipsilateral mouth’s corner upwards and outwards, without the simultaneous activation of other ipsi- or contralateral facial muscles such as mentalis, depressor labii inferioris, depressor anguli oris, platysma, or chewing muscles such as the Masseter. The ZYG response was videorecorded for “off-line” reassessment. The I/t curve was generated in a logarithmic scale by plotting each assessed PW on the *X*-axis and the respective “threshold amplitudes” on the *Y*-axis the [27]. Effects of facial paralysis duration, previous treatments, and etiology on the parameter selection and stimulation performance were evaluated. To determine the safety of the assessed electrostimulation protocol, adverse events were collected and examined.

### 2.8. Parameter/Electrode Position Adjustment

The parameters and the electrode placement chosen during the first stimulation session could be modified at any following visits, in order to maintain a safe and effective ZYG stimulation.

### 2.9. Statistical Analysis

Data were analyzed using IBM SPSS statistics software (Version 25; IBM, New York, NY, USA) for medical statistics. Descriptive statistics were used to report demographic data (e.g., age, gender). Distribution of continuous data was described using mean values with standard deviation. Qualitative data are presented in absolute and relative frequencies.

## 3. Results

### 3.1. ZYG Response to Triangular and Rectangular Wave Stimulation

**Figure 3:**
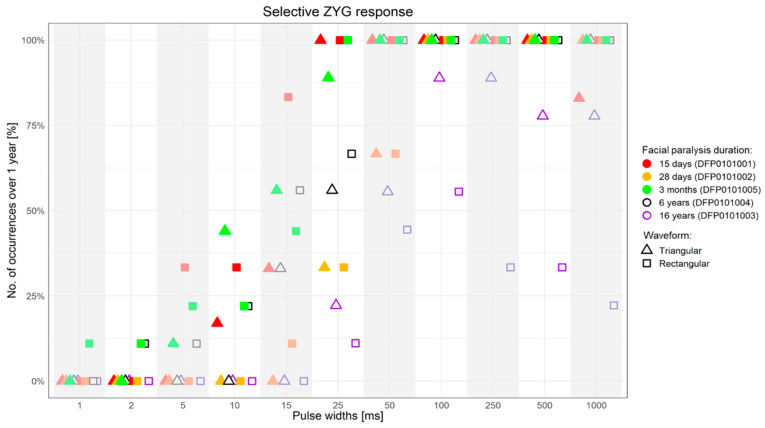
Percentage of successful selective ZYG response (*y*-axis) when the stimulation was presented with specific PWs (*x*-axis) over 1-year follow-up. ms = milliseconds.

**Figure 4:**
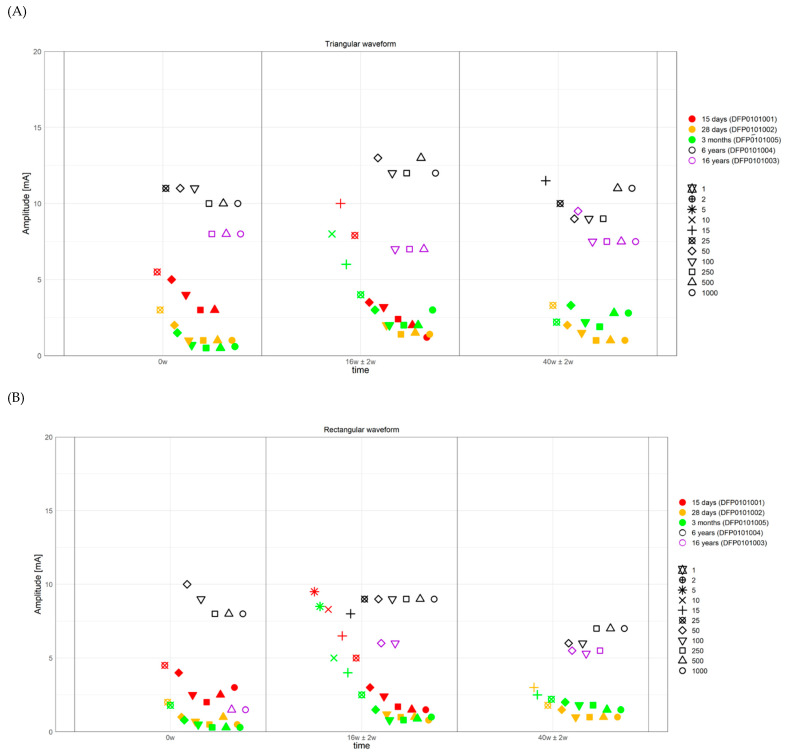
Strength duration curves of all the assessed patients. (**A**) Amplitude required to observe a selective ZYG response (*y*-axis) with triangular waveform stimulation, at selected visits (*x*-axis). (**B**) Amplitude required to observe a selective ZYG response (*y*-axis) with rectangular waveform stimulation, at selected visits (*x*-axis). The colors describe the facial paralysis duration and the form of the plotted outcome the applied PW. ms = milliseconds, mA = milliampere, w = week.

**Figure 5:**
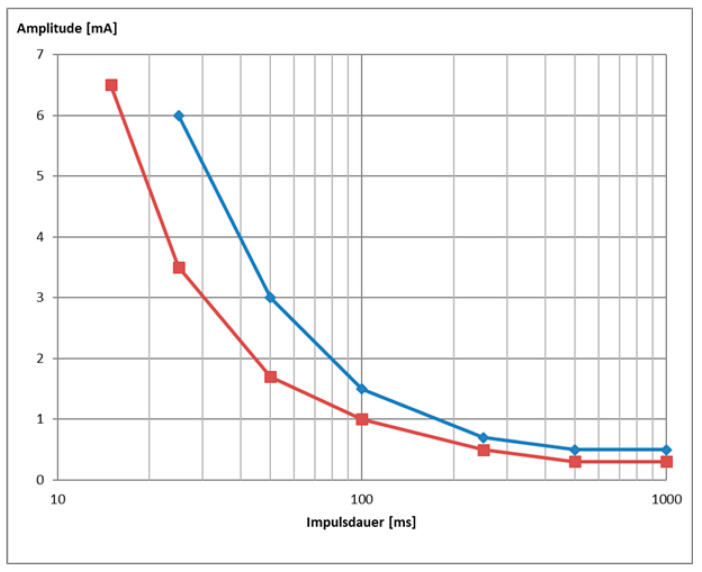
Example of a strength duration curve of patient DFP0101001 at 4 weeks follow up for single pulse stimulation delivered with triangular (blue) or rectangular (red) waveform (data are shown in Figure 6).

**Figure 6:**
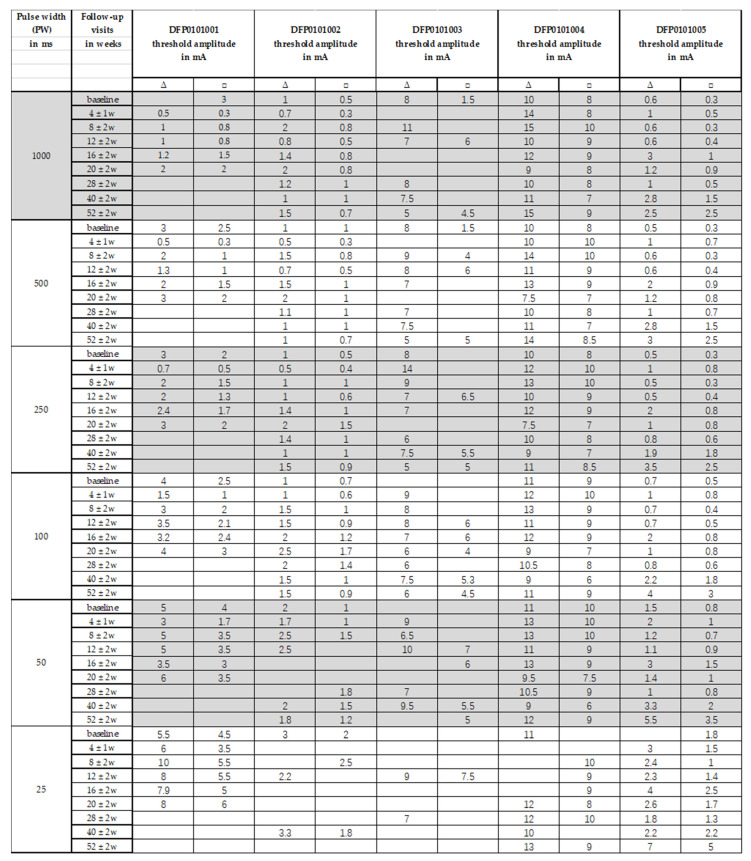
Over time changes in the threshold amplitude required to observe a selective ZYG response without unspecific activation of other facial muscles and/or pain/discomfort for evaluated PWs between 1000 ms and 25 ms with stimulations delivered with a triangular ∆ or a rectangular □ waveform.

The results obtained with triangular and rectangular waveform stimulation were very similar, though with rectangular waveforms the selective ZYG response could be elicited with lower amplitudes at all the assessed PW (Figure 3, Figure 4, Figure 5 and Figure 6). We observed that the duration of facial paralysis affects the excitability of the ZYG, in that patients suffering from facial paralysis for years required higher amplitudes to elicit a specific ZYG response (DFP0101003, −004) irrespectively of the applied PW compared to patients with a fresh paralysis (less than 4 months, DFP0101001, −002, −005).

All the 5 assessed patients, irrespectively of the facial paralysis duration, showed a selective ZYG response when triangular or rectangular stimulation was delivered with a PW ≥ 50 ms (Figure 3 and Figure 4). In patients DFP0101001, −002, and −005, suffering from short-term denervation, the selective ZYG response was observed with amplitudes ≤ 6 mA with triangular waveform and between 0.3 and 4 mA with rectangular waveform, while the same results could be achieved with amplitudes between 5 and 15 mA (triangular) and 1.5 and 10 mA (rectangular) in the patients DFP0101003 and −004, suffering from long term paralysis.

**Figure 7:**
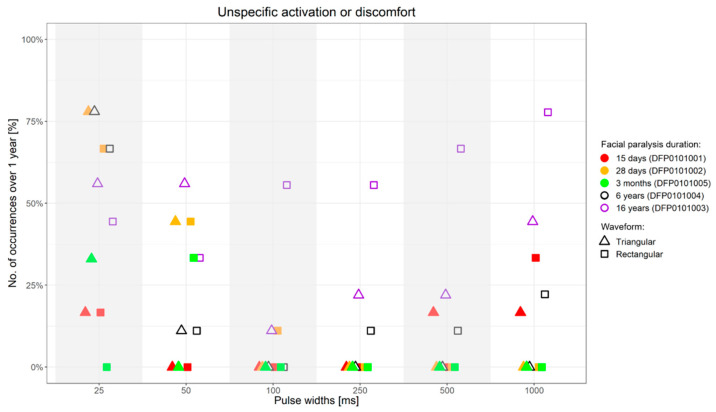
Percentage of occurrence of discomfort or unselective facial muscle response before reaching an amplitude sufficient to elicit a selective ZYG response (*y*-axis) when the stimulation was presented with specific PWs (*x*-axis) over 1-year follow-up upon first stimulation. ms = milliseconds.

Independently from the facial paralysis duration and applied wave form, the amplitude required to elicit a selective ZYG response was found to be inversely proportional to the applied PW (Figure 4). In addition, we found out that within the same patient, the amplitude necessary to observe a selective ZYG response at the various PWs did not remarkably change over time (Figure 6). For stimulations delivered with a PW < 25 ms, either the discomfort threshold was reached, or unselective facial muscle response observed before an amplitude could be applied that triggered a selective ZYG response (unshown data). When a PW of 25 ms was applied, all the patients apart from DFP0101003 (facial paralysis since 16 years), showed a stable selective ZYG response after 6m of stimulation. Prior to this check-point, the discomfort threshold was reached or unselective facial muscle response observed before the selective ZYG response could be elicited (Figure 6). Differently from the other assessed patients, DFP0101003 repeatedly reached the discomfort threshold or showed unselective facial muscle response, before an amplitude could be applied that triggered a selective ZYG response with PWs of 50 and 1000 ms (Figure 7). This effect was more conspicuous with stimulations delivered with rectangular than triangular waveforms.

### 3.2. Use of Trains of Stimulation Bursts

The ZYG response to trains of stimulations was tested at 7 Hz with a PW of 50 ms. Lower PWs were not used, since within single pulse stimulation, they mostly triggered an unselective facial muscle response or caused discomfort to the patient. In general, the threshold amplitude for the trains was lower than that observed for single pulse stimulation and in most cases, it elicited a selective ZYG response only to the first burst, while the subsequent ones did not trigger any ZYG contraction (data not shown). When the threshold amplitude was increased of about 0.5–1 mA above the threshold, the ZYG response was observed for each applied burst. In some cases, the ZYG contraction observed with the first burst was kept through the entire train, suggesting a “tetanic contraction” in response to the delivered train of stimulation. Only for two patients (DFP0101004 and −005), the stimulation delivered in trains was always or almost always effective throughout the entire study follow-up (data not shown). As in the case of single pulse, the train stimulation delivered with triangular was more successful than that delivered with rectangular waveforms, because of the very low discomfort threshold showed by the latter. Similarly, lower amplitudes were needed in patients freshly diagnosed with facial paralysis than in patients suffering from it for years, in order to observe a selective ZYG response (data not shown). Interestingly, for the patient suffering from facial paralysis for about 16 years (DFP0101003), the train stimulation resulted almost always ineffective at either waveform because of the extremely low discomfort threshold showed by this patient (data not shown).

### 3.3. Strength/Duration Curves

The I/t curves (Figure 5) generated with the results of the stimulations delivered between 1 and 1000 ms with triangular and rectangular waveforms were used to preliminary assess the accommodation quotient (AQ), the rheobase, and the chronaxie (data not shown). The results were mixed and due to the reduced sample size we assessed, no clinically relevant conclusions could be drawn based on them.

### 3.4. Side-Effects

For the entire duration of the study, the surface stimulation protocol never associated with the occurrence of serious adverse events. After the stimulation sessions, the skin of the patients showed a transient, slight reddening at the electrode positions, suggested mild irritation. However, burns or other injuries to the skin never occurred. As expected, the electrostimulation did not prevent reinnervation in patients either before or during the participation in this study, independently from the fact that they underwent a reinnervation surgery or not.

## 4. Discussion

In our first attempt to assess SES for the treatment of facial paralysis, we chose the denervated ZYG as main target because of its position sufficiently distant from sensitive spots, such as the skin around the eyes, and the easy detectability of its response by means of mere visual inspection of the ipsilateral mouth corner rising. The unspecific responses of other muscles proximal to the ZYG such as mouth, chin, and chewing muscles, was just as easily noticeable, allowing a quick and effective correction of the stimulation parameters and/or electrode placement during the test session. We chose to evaluate exclusively patients suffering from complete facial paralysis in order to avoid result biases due to reinnervation processes (e.g., ZYG activation due to eye closure in synkinetic reinnervated patients independently from the stimulation), but this meant a rather slow recruitment due to the fact that complete denervated facial paralysis patients are much more seldom than patients suffering from incomplete types of facial palsies.

Single pulse stimulation with PWs ≥ 50 ms, either with triangular or rectangular waveform, is able to induce the selective response of the ZYG, independently from the duration of the stimulation and/or of the facial paralysis, although we noticed a direct association between this latter factor and the amplitude value needed to elicit the selective ZYG response, irrespectively of the applied waveform (Figure 3 and Figure 6).

In particular, while in freshly diagnosed patients an average amplitude ≤ 4 mA sufficed, up to three times this amplitude was required to elicit the selective ZYG response in patients suffering from facial paralysis for more than five years (Figure 3 and Figure 6). This observation seems to confirm previously published results [21,22,23,24,25,26,28] with patients suffering from spinal cord injuries and a complete conus or cauda equina syndrome. In these works, the authors showed that in complete denervated muscles the muscle atrophy progresses always and this reduction is clearly detectable by means of magnetic resonance imaging (MRI). In agreement with these findings, our results suggest that with time patients suffering from complete facial paralysis tend to undergo progressive degradation of the ZYG muscle mass, and loss of contractile properties. This could explain why higher amplitudes are needed to elicit a specific ZYG response in patients suffering from this disease for years rather than months. The MRI pictures let expect that a complete loss of contractible muscle tissue depending on the muscle size as well. Interestingly, while Carraro et al. 2015 [25] described a complete degeneration of human limb muscles after three to six years of denervation, we were able to elicit a contraction of the denervated ZYG in a patient suffering from complete facial paralysis for 16 years. If this result depending on another degeneration pattern of facial compared to limb muscles or of other factors is just speculation.

More intriguingly, in this patient (DFP0101003), triangular waveform stimulation was effective only with a PW of 100 or 250 ms, while in patient DFP0101004 (facial paralysis for 6 years), all the PW ≥ 50 ms were found effective. Whether this difference is due to the fact that DFP0101004 has already undergone SES for two years at the time of enrolment or to the fact that DFP0101003 is suffering from facial paralysis for about thrice the time of DFP0101004 is unclear. Only the recruitment of additional patients with a long history of facial paralysis similar to that of these two patients, will be able to give an accurate answer to these questions.

Our results suggest that the use of rectangular waveform, of PWs < 50 ms, and/or the use of train rather than single pulse stimulation are all factors that may enhance the risk to reach the discomfort threshold and/or cause unspecific activation of other facial muscles or co-contractions before an amplitude could be applied that elicited a selective ZYG response. All the aforementioned factors tend to shorten the stimulation delivery time and/or to increase the applied energy to the target muscle fibers. For instance, the rise of triangular pulses is flatter and the energy applied per pulse is about half of rectangular waveforms (Figure 2), accounting for why stimulation delivered in triangular rather than rectangular waveform is perceived by the patients as more tolerable (Figure 3, Figure 6 and Figure 7). The pulse shape is also likely to explain the need of higher amplitudes we observed with triangular rather than rectangular pulses in order to elicit a selective ZYG response. Despite the higher amplitudes, the transmitted energy is lower for triangular pulses than for rectangular ones. Due to the shortening of the PW, both pulse shapes require higher amplitudes to transfer the same amount of energy to the muscle. The rise for triangular pulses is steeper due to the shortening. Thus, shortening the PW below 50 ms means a faster and more focused transfer of energy to the muscle tissue. Also, when train rather than single pulse stimulation is chosen, a significantly greater amount of energy is transferred in a relatively short time.

A faster and more focused transfer of a greater amount of energy to the muscle increases the electrical field and the recruitment of nerve fibers and innervated muscles, as well as an excessive activation of sensory axons, which in turn decreases the discomfort threshold and increases the likelihood of causing the unselective contraction of other (innervated) facial muscles [29,30]. This can be explained by the different reactions of nerve and muscle fiber membranes. Since nerve membranes can be depolarized by shorter pulses, i.e., fast potential changes, whereas the membranes of denervated muscle fibers respond to “slow” potential changes [31], we were able to stimulate denervated muscles without co-activating sensory axons with PW ≥ 50 ms.

It shall be noted that according to our protocol, when a PW caused discomfort and/or unspecific activation of other facial muscles, shorter PWs were not further tested, in order to avoid extra-burden to the patients. Thus, we did not collect sufficient data for PWs below 25 ms for analysis.

Ravara et al. 2018 and Albertin et al. 2018 [32,33] investigated skin histologic changes upon repeated stimulations in patients suffering from spinal cord injuries and a complete conus or cauda equina syndrome. The patients performed daily at home electrical stimulation of limb muscles with large-area electrodes and amplitudes up to 250 mA for two years. The authors found out that the skin of these patients undergoing repeated stimulation showed increased thickness accompanied by epidermis growth and relevant atrophy reduction. They did not report skin damages following stimulation with the aforementioned parameters. Similarly, we did not observe relevant adverse events caused by the ZYG stimulation, apart from transient, mild reddening of the skin where the electrodes were placed, indicating mild irritation. No burns or other side effects needing medical attention were observed for the entire duration of the study following stimulation.

Finally, it is worth noticing that the SES conducted with our protocol did not associate with delayed or misdirected reinnervation, or chronic pain, for the entire one-year follow-up period assessed in our study, strongly suggesting that SES could become a safe treatment or co-treatment for facial paralysis patients.

## 5. Conclusions

Our preliminary results showed that it is possible to find stimulation parameters able to elicit an effective selective stimulation of a specific facial muscle, in our case, of the ZYG, without causing discomfort to the patient and without causing unwanted unspecific reactions of other ipsi-and/or contralateral facial muscles. We observed that the SES effectiveness is strongly conditioned by the correct electrode placement, which ideally should exclusively interest the area of the target muscle and its immediate proximity. These results suggest that SES could be implemented for facial paralysis therapy, but should be limited to facial muscles the size and position of which would rule out the possibility to place the electrodes off-target and thus elicit a non-specific response of more muscles at the same time. Within these conditions, it is possible to place electrodes and select stimulation parameters in order to obtain a selective, simultaneous or sequential stimulation of both agonistic and antagonistic muscles with the same or different parameters in order to obtain an improved facial mimic. At all events, it should be always considered that the placement of surface electrodes, close to sensitive zones of the face, such as the thin skin around the eyes, could associate with the occurrence of side effects such as reddening and/or burning.

## Figures and Tables

**Table 1:** Patients’ demographic data and facial paralysis history. FP = facial paralysis; F = female; M = male; SR-nEMG time = time during the study follow-up when synkinetic reinnervation signs were detected by means of n-EMG; d = days; m = months; y = years; R = right; L = Left; FNSAMG = Facial nerve suture with auricularis magnus nerve grafting; HFJA = hypoglossus-facial nerve jump anastomosis.

Subject’s Code	Gender	Age	FP Duration	FP Side	FP Origin	FP Surgery	FP Surgery Time	SR-nEMG Time
DFP0101001	F	37	15 d	R	FP after resection of a intraparotideal facial nerve schwannoma treated with direct nerve suture	FNSAMG	2 w before study start	6m
DFP0101002	F	29	28 d	R	Postoperative FP after resection of a vestibular schwannoma	HFJA	8 m after study start	12 m
DFP0101003	F	37	16 y 2 m	R	Postoperative FP after resection of multiple meningiomas of the temporal bone	None	N/A	N/A
DFP0101004	M	59	5 y 11 m	L	Intramastoidal facial nerve schwannoma, schwannoma resection and nerve suture with interposition	HFJA	2 w before study start	8 m (single fiber activity)
DFP0101005	F	53	3 m 24 d	L	Postoperative FP after resection of a vestibular schwannoma	None	N/A	12 m

## Data Availability

The data presented in this article were generated between June 2018 and July 2019 in the Facial Nerve Center of the ENT Department of the Jena University Hospital within a longitudinal, open-label, prospective, monocentric, case-series-based, proof of principle clinical investigation, approved by the ethics committee in 2018 (application number 5505–03/18).
